# Scientists Try to Curb Livestock Emissions

**DOI:** 10.1021/acscentsci.2c01060

**Published:** 2022-09-26

**Authors:** Robin Meadows

Persuading a cow to put its head in a chamber to measure the methane it burps up is easy. All it takes is a sweet treat. But curbing emissions of this potent greenhouse gas from cows, sheep, and other ruminants is much harder. The past 2 years have marked major milestones toward this goal as the world witnessed the first approvals of methane-lowering feed additives for livestock.

**Figure:**
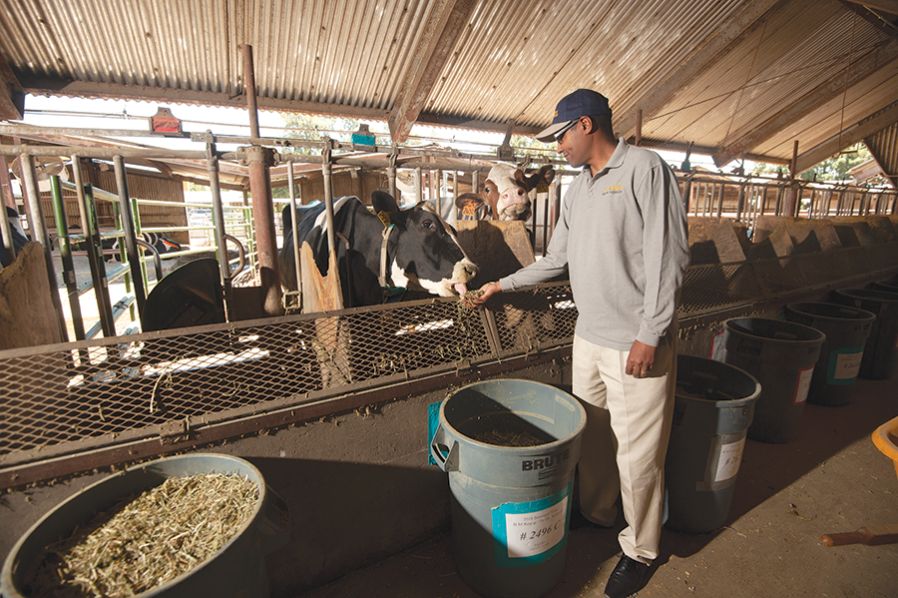
Ermias Kebreab investigated whether adding a small amount of seaweed to cattle feed could reduce methane emissions of these dairy cows. Credit: Gregory Urquiaga/UC Davis

Methane is a major cause of climate change. It’s the second biggest contributor, after carbon dioxide, and responsible for about half a degree of warming, says Andy Reisinger, principal scientist for climate change at New Zealand’s Ministry for the Environment and a member of the Intergovernmental Panel on Climate Change (IPCC) Bureau. The IPCC estimates that human activity accounts for a net temperature increase of 1.1 °C, summing the contributions of greenhouse gas warming with the cooling caused by human-made aerosols.

A single cow emits up to 100 kg of methane per year, and the 3.5 billion ruminants raised as livestock worldwide generate about 6% of anthropogenic greenhouse gas emissions, according to the Food and Agriculture Organization of the United Nations. While this contribution may not seem big enough to focus on, it is high relative to many other economic sectors. “If you argue that a small sector won’t make a difference, then none of them would do anything,” Reisinger says. For comparison, he adds, global aviation is responsible for 2.5% of greenhouse gas emissions, and the airline industry is often targeted for emission reductions.

**Figure:**
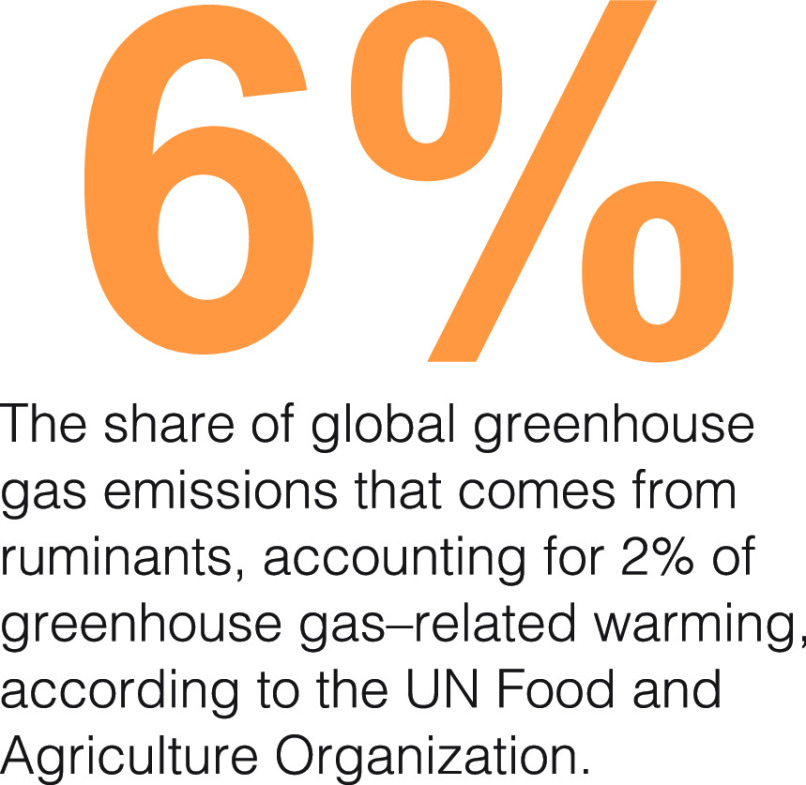
Methane causes one-third of the warming from anthropogenic greenhouse gases. Credit: IPCC

Environmental scientists and farmers have a role in addressing climate change by tracking emissions and implementing solutions, respectively. Animal nutrition scientists play another key part by developing feed additives that suppress ruminant methane production. Their work is beginning to pay off. In February, the European Union joined Chile and Brazil in approving 3-nitrooxypropanol (3-NOP), a molecule that tamps down methane production in ruminants. And in May, California approved a feed additive derived from red seaweed for the same purpose. Scientists are also trying to deliver these molecular tools in longer-lasting forms, which would be easier to use. “It’s unambiguous that we should reduce livestock methane—we need to pull out all the stops for all sectors,” Reisinger says.

The quest to reduce ruminant methane emissions began more than a century ago and was initially driven by hopes of increasing livestock productivity. When livestock eat, some of the energy in their food doesn’t get converted into milk or meat; instead, it gets trapped in the carbon–hydrogen bonds of methane. “Years ago, they would stick an animal in a room and measure all those losses,” says Karen Beauchemin, a ruminant nutrition scientist who recently retired from Agriculture and Agri-Food Canada.

This early work revealed that 4–10% of the energy in livestock feed never even made it to a ruminant’s stomach. That portion of feed was basically wasted in the rumen, the part of the animal’s digestive system that comes right after the esophagus. The rumen is essentially a gigantic fermentation vat where anaerobic microbes break down cellulose from plant cell walls, a process that forms propionate and other volatile fatty acids that serve as ruminants’ primary energy source.

These microbes include archaea that combine hydrogen and CO_2_—two by-products of the rumen’s fermentation—to form the methane that flows from ruminants’ mouths. “It’s constant,” says Ermias Kebreab, a sustainable agriculture scientist at the University of California, Davis. “They’re burping all the time.” By consuming hydrogen, the methanogenic archaea indirectly decrease production of propionate and, in turn, the production of milk and meat.

In the early 2000s, concern about the microbes’ effects qon methane formation shifted from livestock productivity to climate change. Researchers at Royal DSM, a Dutch company specializing in animal nutrition products, began using computer-based studies in their hunt for chemicals that interfere with archaea’s methane-making machinery. “They were looking for molecules similar to a cofactor required by methyl-coenzyme M reductase, the enzyme involved in the last step of methanogenesis,” says Beauchemin, who was not involved in but is familiar with this proprietary work.

The DSM team wondered whether this enzyme, known as MCR, was so distinct from other microbes’ enzymes that a feed additive could limit MCR’s methane production without disrupting other parts of digestion. The team identified 3-NOP, the methanogenesis inhibitor recently approved in the EU, as a likely match for this cofactor. The company then confirmed that 3-NOP could inhibit methane formation in rumen archaea cultured in the lab. Next came successful animal trials. Many researchers worldwide have now established that feeding 3-NOP to livestock reliably decreases rumen methane emissions by 20–40%.

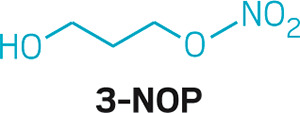



Beauchemin and colleagues extended this body of work to the real world last year by testing 3-NOP in a commercial beef feedlot. Previous studies of this methanogenesis inhibitor in beef cattle had used animals housed individually or in groups of 10 or less. In contrast, the feedlot held more than 4,000 cattle in pens with up to 300 head.

**Figure:**
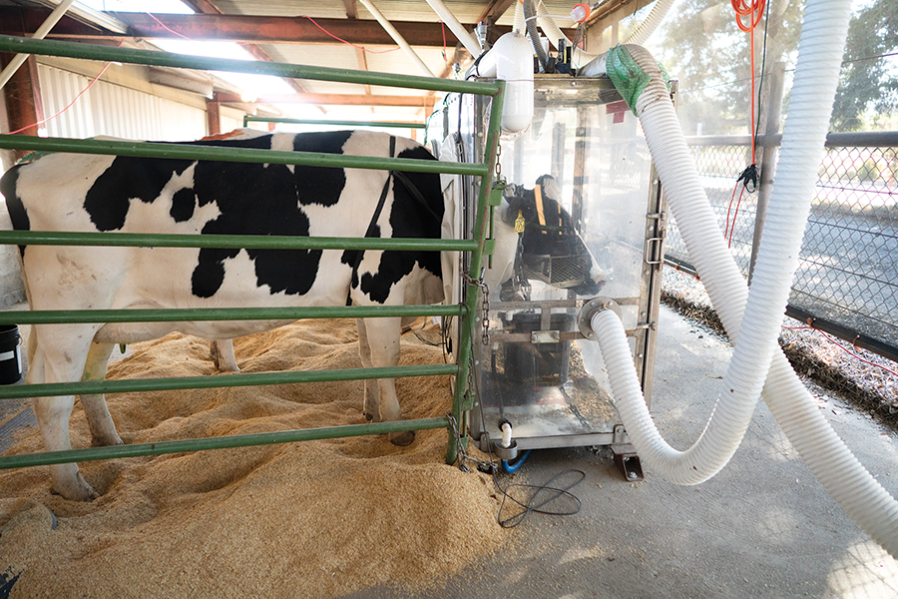
Head-box respiration chambers at the University of California, Davis, capture methane-rich burps from individual cows to test the impact of feed containing red seaweed on emissions. Credit: Karin Higgins/UC Davis

“They compete for food; there’s one long feeding trough,” Beauchemin says. “You don’t know how much each of them ate.” So emissions on a feedlot scale need to be measured in aggregate. Beauchemin’s team did that by beaming lasers across a cow pen and recording what was reflected back. “Methane in the air affects the wavelength that comes back,” she says. “You do it over the control and 3-NOP pens to see the difference.” The study showed that 3-NOP reduced rumen methane emissions up to 26%, on par with previous findings (*Front. Anim. Sci.***2021**, DOI: 10.3389/fanim.2021.641590).

Other researchers are exploring whether 3-NOP can tweak the rumen microbiome more permanently. Their ideas center on arresting microbiome development during a critical window early in life in hopes of reducing emissions from older animals. “Farmers could save quite a bit of money if they could use an inhibitor for a few weeks and then stop and still see the effect later,” says Diego Morgavi, an animal nutrition scientist and microbiologist at the French National Research Institute for Agriculture, Food and Environment.

Calves don’t start burping up methane until they begin the transition to solid food, but methanogens are detected in their rumen the first day of life. “After calves are born, the rumen starts acquiring microbes from the first second,” says Morgavi, who was a large-animal veterinarian before going into research. By 2 weeks old, a calf’s rumen has about as many archaea as an adult’s. At 11 weeks, when calves wean and switch entirely to solid food, methane emissions spike.

Morgavi and colleagues took advantage of this window of opportunity between birth and weaning. They fed calves 3-NOP every day for the first 14 weeks. A year into the study, 3-NOP’s lingering impact left Morgavi’s team surprised “but in a good way,” he says with a smile: they found rumen methane emissions reduced by over 17% (*Sci. Rep.***2021**, DOI: 10.1038/s41598-021-82084-9). While cautioning that this finding needs to be repeated by other groups, Morgavi is eager to test whether 3-NOP early in life can lower methane emissions even longer.

Beyond synthetic molecules, the search for methanogenesis inhibitors has entailed screening thousands of plant materials in cultured rumen archaea. The red seaweed *Asparagopsis taxiformis*, recently approved in California as a feed additive, was a star performer.

In nature, this marine algae produces bromoform and other halogenated compounds to deter sea creatures from eating it. And like 3-NOP, bromoform inhibits the last enzyme in archaeal methanogenesis.

UC Davis’s Kebreab and colleagues found that red seaweed cut rumen methane emissions up to 67% in dairy cows and up to 80% in beef cattle (*J. Cleaner Prod.***2019**, DOI: 10.1016/j.jclepro.2019.06.193; *PLOS One***2021**, DOI: 10.1371/journal.pone.0247820). But because bromoform is a probable human carcinogen, its use raises the question of health effects. The researchers found that bromoform concentrations were not significantly different in the milk of treated and untreated cows, and they detected no bromoform in the meat of treated beef cattle.

Kebreab is particularly excited that red seaweed not only reduced rumen methane emissions but also increased the weight cows put on per kilogram of feed—a metric called feed conversion efficiency—in beef cattle by as much as 14%. “The biggest thing is that the efficiency is huge. We’ve never seen this before,” he says. “This could be a good selling point for farmers.”

Without this increase in feed conversion efficiency, red seaweed would just be an additional cost to be absorbed by farmers, possibly limiting its adoption. But with higher feed conversion efficiency, Kebreab estimates that—depending on the seaweed dose—beef producers could reduce feed costs by $40,000–$87,000 per 1,000 head of cattle.

But these feed-based interventions currently work only on livestock kept in confinement, where farmers formulate the animals’ diet and can easily provide a daily dose of these additives. Many ruminants are raised in pastoral systems, in which they roam and graze freely. “In the high country, sheep flocks may not be seen for months,” says Reisinger, who is also a former deputy director at the New Zealand Agricultural Greenhouse Gas Research Centre, which operates a program dedicated to reducing livestock methane emissions. The country has one of the highest sheep populations per capita in the world, and livestock generate more than 85% of its methane emissions.

Researchers are looking at approaches like slow-release formulations of methanogenesis inhibitors, which farmers could administer less frequently. Another approach is selectively breeding livestock that emit less methane or that produce milk for more of the year. If dairy cows were bred to produce milk for 9 months instead of 6, for example, a smaller herd would produce the same amount of milk with less methane.

Then there’s what Reisinger calls a holy grail of ruminant methane control: a vaccine against the microbes that produce this greenhouse gas. Researchers in New Zealand have developed an antibody that reduces methane in simulated rumen fluid in test tubes as well as in cultured rumen microbes. In addition, vaccinated animals produce this antibody in their saliva, and it reaches their rumens—but the desired outcome of lower methane emissions remains elusive. “The individual elements are all there,” Reisinger says. “They just haven’t shown that it works in live animals.”

Livestock methane emissions are projected to rise 30% by 2050 as the human population produces and consumes increasing amounts of meat and dairy products. Livestock are essential to food security and childhood nutrition in some parts of the world. But high-income populations could lower demand by choosing to eat less animal protein and wasting less food. Reducing overconsumption of animal products would benefit human and ecological health, Reisinger says. “If we take climate change seriously, we need to focus on technological approaches and on demand.”

*Robin Meadows is a freelance contributor to**Chemical & Engineering News**, an independent news publication of the American Chemical Society.*

